# Distinct renal adverse event profiles among CDK4/6 inhibitors: a comparative pharmacovigilance analysis

**DOI:** 10.3389/fphar.2026.1917719

**Published:** 2026-09-17

**Authors:** Suleyman Sami Guzel, Aydan Mutis Alan, Kubilay Tay, Nebi Serkan Demirci, Ozkan Alan

**Affiliations:** 1 Division of Medical Oncology, Department of Internal Medicine, Cerrahpasa Faculty of Medicine, Istanbul University-Cerrahpasa, Istanbul, Türkiye; 2 Division of Nephrology, Department of Internal Medicine, Kartal City Hospital, Istanbul, Türkiye

**Keywords:** breast cancer, CDK4/6 inhibitors, FAERS, pharmacovigilance, renal adverse events, acute kidney injury

## Abstract

**Objective:**

Renal adverse events associated with cyclin-dependent kinase 4 and 6 (CDK4/6) inhibitors have gained increasing attention in clinical practice. However, comparative real-world data regarding renal safety profiles among individual CDK4/6 inhibitors remain limited. This study aimed to characterize renal adverse event reporting associated with abemaciclib, palbociclib, and ribociclib and compare disproportionality signals among these agents using the U.S. Food and Drug Administration Adverse Event Reporting System (FAERS).

**Methods:**

A retrospective pharmacovigilance analysis was conducted using FAERS reports submitted between January 2014 and March 2026. Renal adverse events were identified using Medical Dictionary for Regulatory Activities (MedDRA) Preferred Terms within the Renal and Urinary Disorders System Organ Class. Reporting odds ratios (RORs) with 95% confidence intervals (CIs) were calculated to evaluate disproportionality signals for selected renal and electrolyte adverse events.

**Results:**

Following deduplication, 14,193 reports associated with abemaciclib, 91,359 with palbociclib, and 29,488 with ribociclib were identified. Renal adverse events were reported in 898, 2,636, and 1,826 reports, respectively. Acute kidney injury was more frequently reported with abemaciclib (35.7%) and ribociclib (19.7%) than with palbociclib (12.4%) (p < 0.001). Renal impairment/failure was the most commonly reported grouped renal adverse event category. Abemaciclib demonstrated the highest reporting odds for acute kidney injury (ROR 4.06, 95% CI 3.53–4.67), followed by ribociclib (ROR 1.94, 95% CI 1.69–2.22), whereas ribociclib showed the strongest disproportionality signals for decreased glomerular filtration rate, hypocalcemia, creatinine increased, hyponatremia, and hypokalemia. Overall, 65.6% of reports were serious and hospitalization occurred in 32.5% of cases.

**Conclusion:**

This large-scale pharmacovigilance analysis identified substantial differences in renal adverse event reporting patterns among CDK4/6 inhibitors. Abemaciclib was more frequently associated with acute kidney injury and adverse events suggestive of intrinsic renal injury, whereas ribociclib demonstrated stronger signals for laboratory indicators of renal dysfunction and electrolyte abnormalities. Although spontaneous reporting data cannot establish causality or estimate incidence, these findings suggest heterogeneous renal adverse event profiles among CDK4/6 inhibitors and support careful renal monitoring during treatment.

## 1 Introduction

Cyclin-dependent kinase 4 and 6 (CDK4/6) inhibitors have revolutionized the treatment of hormone receptor-positive (HR-positive), human epidermal growth factor receptor 2-negative (HER2-negative) breast cancer. By inhibiting phosphorylation of the retinoblastoma protein and inducing G1 cell-cycle arrest, palbociclib, ribociclib, and abemaciclib significantly improve progression-free and overall survival outcomes and have become standard components of treatment in both metastatic and selected early-stage settings (Finn et al., 2016; Hortobagyi et al., 2016; Johnston et al., 2020; Hortobagyi et al., 2018; Slamon et al., 2018; Tripathy et al., 2018; Sledge et al., 2020; Rastogi et al., 2024).

Although CDK4/6 inhibitors are generally well tolerated, they are associated with a distinct spectrum of adverse events. Hematologic toxicities, particularly neutropenia, are more frequently observed with palbociclib and ribociclib, whereas gastrointestinal adverse events are more common with abemaciclib. In recent years, increasing attention has focused on renal adverse events associated with CDK4/6 inhibitor therapy. Elevations in serum creatinine are commonly observed during treatment, particularly with abemaciclib, raising concerns regarding potential nephrotoxicity and the need for careful renal monitoring (Chappell et al., 2019).

The renal effects of CDK4/6 inhibitors are complex and not fully understood. Experimental and clinical studies have demonstrated that abemaciclib inhibits renal tubular transporters, including organic cation transporter 2 (OCT2) and multidrug and toxin extrusion transporters (MATE1 and MATE2-K), leading to reversible increases in serum creatinine without affecting measured glomerular filtration rate (Chappell et al., 2019). Consequently, some renal laboratory abnormalities observed during treatment may reflect alterations in creatinine handling rather than true kidney injury. Similar phenomena have been described with several targeted anticancer therapies and may result in so-called “pseudo-acute kidney injury”, potentially complicating the interpretation of renal function during treatment (Vanhoutte and Sprangers, 2023).

Nevertheless, accumulating evidence suggests that clinically significant nephrotoxicity may also occur during CDK4/6 inhibitor therapy. In a clinicopathologic study of biopsy-confirmed cases, Gupta et al. identified acute tubular injury, acute tubular necrosis, and interstitial nephritis among patients who developed acute kidney injury while receiving CDK4/6 inhibitors (Gupta et al., 2022). Additional observational studies have reported declines in estimated glomerular filtration rate, renal impairment, and other kidney-related adverse outcomes, suggesting that the renal safety profile of these agents extends beyond isolated creatinine elevations (Hanna et al., 2023; Cui et al., 2024).

Despite increasing recognition of renal adverse events, available evidence remains limited. Most data originate from clinical trials, retrospective cohorts, or isolated case reports, and direct comparisons among individual CDK4/6 inhibitors are scarce. Furthermore, rare renal toxicities may be underrepresented in clinical trials because of limited sample sizes, restrictive eligibility criteria, and relatively short follow-up periods. Consequently, the comparative renal adverse event reporting profiles of abemaciclib, palbociclib, and ribociclib remain incompletely characterized.

Large pharmacovigilance databases provide an opportunity to evaluate uncommon but clinically relevant toxicities in real-world populations. The U.S. Food and Drug Administration Adverse Event Reporting System (FAERS) is one of the largest spontaneous reporting databases worldwide and has been widely used to identify emerging safety signals associated with anticancer therapies (Noguchi et al., 2021). Therefore, we conducted a comprehensive pharmacovigilance analysis using the FAERS database to characterize renal adverse event reporting associated with abemaciclib, palbociclib, and ribociclib. We aimed to evaluate the spectrum of reported renal toxicities, compare reporting patterns among individual CDK4/6 inhibitors, and assess disproportionality signals for major renal adverse event categories. Improved understanding of these reporting patterns may facilitate earlier recognition and more effective monitoring of renal toxicities during CDK4/6 inhibitor therapy and help generate hypotheses for future prospective studies.

## 2 Materials and methods

### 2.1 Data source and study design

A retrospective pharmacovigilance study was conducted using data obtained from the U.S. Food and Drug Administration (FDA) Adverse Event Reporting System (FAERS), a publicly accessible spontaneous reporting database that collects adverse event reports from healthcare professionals, consumers, and pharmaceutical manufacturers (Potter et al., 2025). FAERS reports submitted between January 2014 and March 2026 were retrieved and evaluated.

The analysis focused on the three currently approved cyclin-dependent kinase 4 and 6 (CDK4/6) inhibitors used for breast cancer treatment: abemaciclib, palbociclib, and ribociclib. Adverse event reports were identified using generic drug names and extracted from the FAERS Public Dashboard. Case retrieval was performed using the active ingredient (generic name) search function of the FAERS Public Dashboard. This function applies the standardised drug nomenclature maintained by the FDA and maps reported trade names (Verzenio for abemaciclib, Ibrance for palbociclib and Kisqali for ribociclib), synonyms, abbreviations and common spelling variants to the corresponding active ingredient. Reports were therefore retrieved irrespective of whether the product had been recorded under its generic or recognized brand name in the original submission, and no additional manual brand-name mapping was performed. Reports in which the CDK4/6 inhibitor was designated as the primary suspected (PS) or secondary suspected (SS) drug were included in the analysis. Reports were retrieved at the case level, and duplicate records were removed according to FDA recommendations. When multiple reports shared the same case identification number, only the most recent and most complete report was retained for analysis.

### 2.2 Case identification and MedDRA coding

Renal adverse events were identified using Preferred Terms (PTs) included within the Medical Dictionary for Regulatory Activities (MedDRA version 28.0) (International Council for Harmonisation of Technical Requirements for Pharmaceuticals for Human Use, 2025) Renal and Urinary Disorders System Organ Class (SOC). All renal-related PTs reported in association with abemaciclib, palbociclib, or ribociclib were extracted and reviewed.

For descriptive analyses, individual PTs were grouped into clinically relevant renal adverse event categories, including acute kidney injury (AKI), creatinine increased, renal impairment/failure, acute tubular necrosis (ATN), tubulointerstitial nephritis (TIN), renal tubular disorders, glomerulonephritis, and proteinuria. A report could contribute to more than one category if multiple renal PTs were reported within the same case. For grouped adverse event categories, a report was counted only once within a given category, regardless of the number of relevant MedDRA preferred terms reported in the same case. Therefore, category counts represent unique reports and may differ from the arithmetic sum of individual preferred-term frequencies presented in Supplementary Table S1. The assignment of individual PTs to grouped renal adverse event categories was performed by the investigators based on clinical relevance and nephrology literature.

A total of 17,654 reports associated with abemaciclib, 91,661 reports associated with palbociclib, and 29,878 reports associated with ribociclib were initially identified. After removal of duplicate records, 14,193 abemaciclib reports, 91,359 palbociclib reports, and 29,488 ribociclib reports remained for analysis. Among these, renal adverse events were identified in 898 reports associated with abemaciclib, 2,636 reports associated with palbociclib, and 1,826 reports associated with ribociclib.

The complete list of MedDRA Preferred Terms used for case identification is provided in Supplementary Table S1. For each preferred term, the number of reports is additionally expressed as a proportion of all deduplicated reports submitted for the corresponding drug (abemaciclib, n = 14,193; palbociclib, n = 91,359; ribociclib, n = 29,488), to facilitate descriptive comparison across agents while accounting for differences in overall reporting volume.

### 2.3 Data collection and clinical characteristics

Demographic and clinical variables extracted from FAERS included age, sex, year of reporting, geographic region, seriousness classification, and reported clinical outcomes. Seriousness outcomes were defined according to FDA criteria and included death, hospitalization, disability, life-threatening events, and other serious outcomes.

Reports were categorized according to the suspected CDK4/6 inhibitor, and descriptive analyses were performed to characterize differences in reporting patterns among abemaciclib, palbociclib, and ribociclib. Because a single FAERS report may contain more than one reported outcome, individual outcome categories were not mutually exclusive.

### 2.4 Disproportionality analysis

Disproportionality analyses were performed using reporting odds ratios (RORs) and corresponding 95% confidence intervals (CIs) to evaluate the association between individual CDK4/6 inhibitors and selected renal adverse events.

For each analysis, reports involving one CDK4/6 inhibitor were compared with reports involving the other two CDK4/6 inhibitors combined, which served as the reference group. This comparator strategy was selected to reduce confounding related to indication, treatment setting, and patient characteristics that may arise when using the entire FAERS database as a reference population. Cases were defined as reports containing the renal adverse event of interest, whereas non-cases comprised all other reports associated with CDK4/6 inhibitors that did not include the specified event. The case definitions applied in the disproportionality analysis were narrower than the grouped categories summarised in Table 2. Acute kidney injury, creatinine increased and decreased glomerular filtration rate were each defined by the corresponding single MedDRA Preferred Term, whereas renal impairment/failure combined the Preferred Terms renal impairment and renal failure, counted only once per report. Case counts in Table 3 therefore differ from the grouped category counts in Table 2, which additionally include related Preferred Terms.

RORs were calculated using standard 2 × 2 contingency tables. A disproportionality signal was considered present when the lower bound of the 95% confidence interval exceeded 1.0. Because FAERS is a spontaneous reporting system and does not provide reliable exposure data, RORs were interpreted as measures of disproportional reporting rather than estimates of incidence or relative risk.

### 2.5 Statistical analysis

Descriptive statistics were used to summarize demographic characteristics, reporting patterns, and clinical outcomes. Categorical variables were compared using the chi-square test, whereas continuous variables were compared using the Kruskal–Wallis test because age distributions were non-normally distributed.

Results were expressed as frequencies, percentages, medians, and interquartile ranges (IQRs), as appropriate. Given the spontaneous reporting nature of FAERS and the absence of reliable exposure denominators, all p-values were considered descriptive and should not be interpreted as measures of causality, incidence, or relative risk. Statistical analyses were performed using IBM SPSS Statistics for Windows, version 29 (IBM Corp., Armonk, NY, United States).

## 3 Results

### 3.1 Patient characteristics

A total of 5,360 renal adverse event reports associated with CDK4/6 inhibitors were identified in the FAERS database, including 898 reports associated with abemaciclib, 2,636 reports associated with palbociclib, and 1,826 reports associated with ribociclib.

Female patients accounted for the majority of reports (92.1%), whereas male patients represented 2.3%; sex was not specified in 5.6% of cases. The median patient age was 63 years (IQR 57–70), and 63.2% of patients were aged 65 years or older. Age distributions differed significantly among CDK4/6 inhibitors, with a greater proportion of patients aged ≥65 years among palbociclib-associated reports compared with abemaciclib- and ribociclib-associated reports (p < 0.001).

Most reports originated from North America (52.0%), followed by Europe (32.3%) and Asia (9.6%). Regarding reporting period, 39.3% of reports were submitted between 2018 and 2022, whereas 16.7% were reported between 2023 and 2026. Earlier reports (<2018) accounted for 8.1% of cases, while reporting year was unavailable in 35.9%.

Overall, 65.6% of renal adverse event reports were classified as serious. Significant differences were observed among CDK4/6 inhibitors with respect to sex distribution, age group, reporting year, geographic region, and reported clinical outcomes (all p < 0.001). Patient demographics and reporting characteristics are summarized in Table 1.

**TABLE 1:** Demographic and clinical characteristics of renal adverse event reports associated with CDK4/6 inhibitors.

Characteristic	Whole cohort n = 5360	Abemaciclib n = 898	Palbociclib n = 2636	Ribociclib n = 1826	p-value
Sex	​	​	​	​	<0.001
Female	4937 (92.1)	780 (86.9)	2424 (92.0)	1733 (94.9)	​
Male	124 (2.3)	32 (3.6)	69 (2.6)	23 (1.3)	​
Not specified	299 (5.6)	86 (9.6)	143 (5.4)	70 (3.8)	​
Age group	​	​	​	​	<0.001
≥65 years	2322 (63.2)	375 (58.3)	1512 (66.8)	435 (56.4)	​
<65 years	1354 (36.8)	268 (41.7)	750 (33.2)	336 (43.6)	​
Age (years)	​	​	​	​	<0.001†
Median (IQR)	63 (57–70)	61 (54–69)	64 (58–71)	65 (59–72)	​
Report year	​	​	​	​	<0.001
<2018	432 (8.1)	2 (0.2)	385 (14.6)	45 (2.5)	​
2018–2022	2109 (39.3)	256 (28.5)	1000 (37.9)	853 (46.7)	​
2023–2026	895 (16.7)	249 (27.7)	263 (10.0)	383 (21.0)	​
Unknown	1924 (35.9)	391 (43.6)	988 (37.5)	545 (29.8)	​
Continent	​	​	​	​	<0.001
North America	2785 (52.0)	411 (45.8)	1376 (52.2)	998 (54.7)	​
Europe	1732 (32.3)	302 (33.6)	828 (31.4)	602 (33.0)	​
Asia	512 (9.6)	118 (13.1)	263 (10.0)	131 (7.2)	​
South America	188 (3.5)	41 (4.6)	87 (3.3)	60 (3.3)	​
Oceania	54 (1.0)	9 (1.0)	26 (1.0)	19 (1.0)	​
Other	89 (1.6)	17 (1.9)	56 (2.1)	16 (0.9)	​
Seriousness	​	​	​	​	0.42
Serious	3514 (65.6)	583 (64.9)	1718 (65.2)	1213 (66.4)	​
Not serious	1846 (34.4)	315 (35.1)	918 (34.8)	613 (33.6)	​
Outcomes	​	​	​	​	<0.001
Death	642 (12.0)	121 (13.5)	316 (12.0)	205 (11.2)	​
Hospitalization	1743 (32.5)	297 (33.1)	847 (32.1)	599 (32.8)	​
Disability	217 (4.0)	42 (4.7)	104 (3.9)	71 (3.9)	​
Life-threatening	389 (7.3)	78 (8.7)	187 (7.1)	124 (6.8)	​
Other/Unknown	2369 (44.2)	360 (40.1)	1182 (44.9)	827 (45.3)	​

Values are presented as n (%) unless otherwise stated. †Kruskal–Wallis test for age; chi-square test for categorical variables. IQR, interquartile range. All p-values are descriptive only.

### 3.2 Spectrum of renal adverse events

A broad spectrum of renal adverse events was identified across the three CDK4/6 inhibitors. Among individual MedDRA Preferred Terms, renal impairment was the most frequently reported event (n = 1,107), followed by acute kidney injury (n = 917), renal failure (n = 834), renal disorder (n = 507), and blood creatinine increased (n = 434).

Other commonly reported events included nephrolithiasis (n = 243), chronic kidney disease (n = 202), glomerular filtration rate decreased (n = 137), hydronephrosis (n = 127), renal cyst (n = 80), renal pain (n = 73), nephropathy toxic (n = 72), and renal injury (n = 48). Less frequent reported adverse events included renal tubular necrosis (n = 42), tubulointerstitial nephritis (n = 37), proteinuria (n = 29), anuria (n = 22), and pyelonephritis (n = 18).

The distribution of individual renal adverse event preferred terms is presented in Supplementary Table S1. Because the total number of reports differed substantially between agents, preferred-term counts in Supplementary Table S1 are also presented as proportions of all deduplicated reports for the corresponding drug. Expressed in this way, acute kidney injury was recorded in 2.064% of all abemaciclib reports, 0.332% of all palbociclib reports and 1.089% of all ribociclib reports, whereas blood creatinine increased was recorded in 0.578%, 0.148% and 0.736% of reports, respectively.

### 3.3 Grouped renal adverse event categories

The distribution of grouped renal adverse event categories differed significantly among individual CDK4/6 inhibitors. Renal impairment/failure represented the most frequently reported grouped category across all agents, accounting for 34.0% of renal adverse event reports associated with abemaciclib, 36.8% with palbociclib, and 43.5% with ribociclib (p < 0.001).

Acute kidney injury (AKI) was reported more frequently with abemaciclib (35.7%) than with palbociclib (12.4%) or ribociclib (19.7%) (p < 0.001). Similarly, acute tubular necrosis (ATN) was observed more frequently among abemaciclib-associated reports (2.3%) compared with palbociclib (0.3%) and ribociclib (0.7%) reports (p < 0.001).

Creatinine increased was reported in 9.8% of abemaciclib-associated renal adverse event reports, 6.2% of palbociclib-associated reports, and 12.8% of ribociclib-associated reports (p < 0.001). Tubulointerstitial nephritis (TIN) occurred more frequently with abemaciclib (1.3%) than with palbociclib (0.5%) or ribociclib (0.6%) (p = 0.036).

Renal tubular disorders were uncommon overall but were reported more frequently with abemaciclib than with the other CDK4/6 inhibitors (p = 0.007). No statistically significant differences were observed among treatment groups for glomerulonephritis or proteinuria.

Grouped renal adverse event categories are summarized in Table 2.

**TABLE 2:** Distribution of grouped renal adverse event categories across CDK4/6 inhibitors.

Grouped renal AE category	Abemaciclib n (%) (n = 898)	Palbociclib n (%) (n = 2636)	Ribociclib n (%) (n = 1826)	p-value
Acute kidney injury (AKI)	321 (35.7)	326 (12.4)	360 (19.7)	<0.001
Creatinine increased	88 (9.8)	164 (6.2)	234 (12.8)	<0.001
Renal impairment/failure	305 (34.0)	969 (36.8)	794 (43.5)	<0.001
Acute tubular necrosis (ATN)	21 (2.3)	8 (0.3)	13 (0.7)	<0.001
Tubulointerstitial nephritis (TIN)	12 (1.3)	14 (0.5)	11 (0.6)	0.036
Renal tubular disorders	5 (0.6)	3 (0.1)	1 (0.1)	0.007
Glomerulonephritis	14 (1.6)	22 (0.8)	15 (0.8)	0.121
Proteinuria	9 (1.0)	11 (0.4)	9 (0.5)	0.112

Values are presented as n (%). Multiple categories can be present in the same report; percentages do not sum to 100%.

### 3.4 Clinical outcomes

Among all renal adverse event reports, hospitalization was reported in 32.5% of cases, followed by death (12.0%), life-threatening events (7.3%), and disability (4.0%).

Reported outcome distributions differed significantly among CDK4/6 inhibitors (p < 0.001). Death was reported in 13.5% of abemaciclib-associated reports, 12.0% of palbociclib-associated reports, and 11.2% of ribociclib-associated reports. Hospitalization occurred in approximately one-third of reports across all treatment groups, ranging from 32.1% to 33.1%.

Despite differences in specific clinical outcomes, no statistically significant differences were observed in overall seriousness classification among the three CDK4/6 inhibitors (p = 0.42), with approximately two-thirds of reports categorized as serious adverse events.

### 3.5 Disproportionality analysis

Disproportionality analyses identified differences in renal adverse event reporting patterns among CDK4/6 inhibitors.

Abemaciclib demonstrated the highest reporting odds for acute kidney injury (ROR 4.06, 95% CI 3.53–4.67), followed by ribociclib (ROR 1.94, 95% CI 1.69–2.22), whereas palbociclib showed markedly reduced reporting odds for this event (ROR 0.23, 95% CI 0.20–0.27).

For laboratory indicators of renal dysfunction and for electrolyte abnormalities, ribociclib demonstrated the strongest disproportionality signals. Increased reporting odds were observed for decreased glomerular filtration rate (ROR 5.19, 95% CI 3.69–7.30), hypocalcemia (ROR 5.21, 95% CI 2.42–11.22), creatinine increased (ROR 3.60, 95% CI 2.98–4.35), hyponatremia (ROR 3.23, 95% CI 2.07–5.04), and hypokalemia (ROR 2.54, 95% CI 1.47–4.39). Ribociclib was additionally associated with increased reporting odds for renal impairment/failure (ROR 2.26, 95% CI 2.06–2.48).

Abemaciclib also showed increased reporting odds for creatinine increased (ROR 1.99, 95% CI 1.56–2.53), renal impairment/failure (ROR 1.57, 95% CI 1.39–1.78) and hypokalemia (ROR 3.36, 95% CI 1.85–6.12), but not for decreased glomerular filtration rate, hyponatremia or hypocalcemia. In the pairwise comparisons, abemaciclib demonstrated stronger reporting associations than either comparator for acute tubular necrosis and tubulointerstitial nephritis. Palbociclib showed reduced reporting odds across all evaluated renal and electrolyte adverse events.

Reporting odds ratios for selected renal and electrolyte adverse events are shown in Table 3, and pairwise comparisons between individual agents are presented in Figure 1. These findings represent disproportionality signals derived from spontaneous reports and should not be interpreted as estimates of incidence or causal risk.

**TABLE 3:** Disproportionality analysis (ROR) of selected renal and electrolyte adverse events among CDK4/6 inhibitors.

Adverse event	Abemaciclib n (%) — ROR (95% CI)	Palbociclib n (%) — ROR (95% CI)	Ribociclib n (%) — ROR (95% CI)
Acute kidney injury	293 (2.06%) 4.06 (3.53–4.67)	303 (0.33%) 0.23 (0.20–0.27)	321 (1.09%) 1.94 (1.69–2.22)
Creatinine increased	82 (0.58%) 1.99 (1.56–2.53)	135 (0.15%) 0.21 (0.18–0.26)	217 (0.74%) 3.60 (2.98–4.35)
Renal impairment/failure	296 (2.09%) 1.57 (1.39–1.78)	882 (0.97%) 0.40 (0.37–0.44)	733 (2.49%) 2.26 (2.06–2.48)
GFR decreased	6 (0.04%) 0.39 (0.17–0.88)	50 (0.05%) 0.27 (0.19–0.39)	81 (0.27%) 5.19 (3.69–7.30)
Hypokalemia	15 (0.11%) 3.36 (1.85–6.12)	16 (0.02%) 0.21 (0.11–0.37)	22 (0.07%) 2.54 (1.47–4.39)
Hyponatremia	7 (0.05%) 0.84 (0.39–1.82)	34 (0.04%) 0.37 (0.24–0.58)	37 (0.13%) 3.23 (2.07–5.04)
Hypocalcemia	3 (0.02%) 1.06 (0.32–3.54)	8 (0.01%) 0.20 (0.09–0.46)	16 (0.05%) 5.21 (2.42–11.22)

n (%) indicates the number and proportion of reports containing the adverse event among all deduplicated reports submitted for the respective drug (abemaciclib, N = 14,193; palbociclib, N = 91,359; ribociclib, N = 29,488). Reporting odds ratios were calculated using the other two CDK4/6 inhibitors combined as the reference group. ROR, reporting odds ratio; CI, confidence interval; GFR, glomerular filtration rate. Signals were considered present when the lower bound of the 95% confidence interval exceeded 1.0.

**FIGURE 1:**
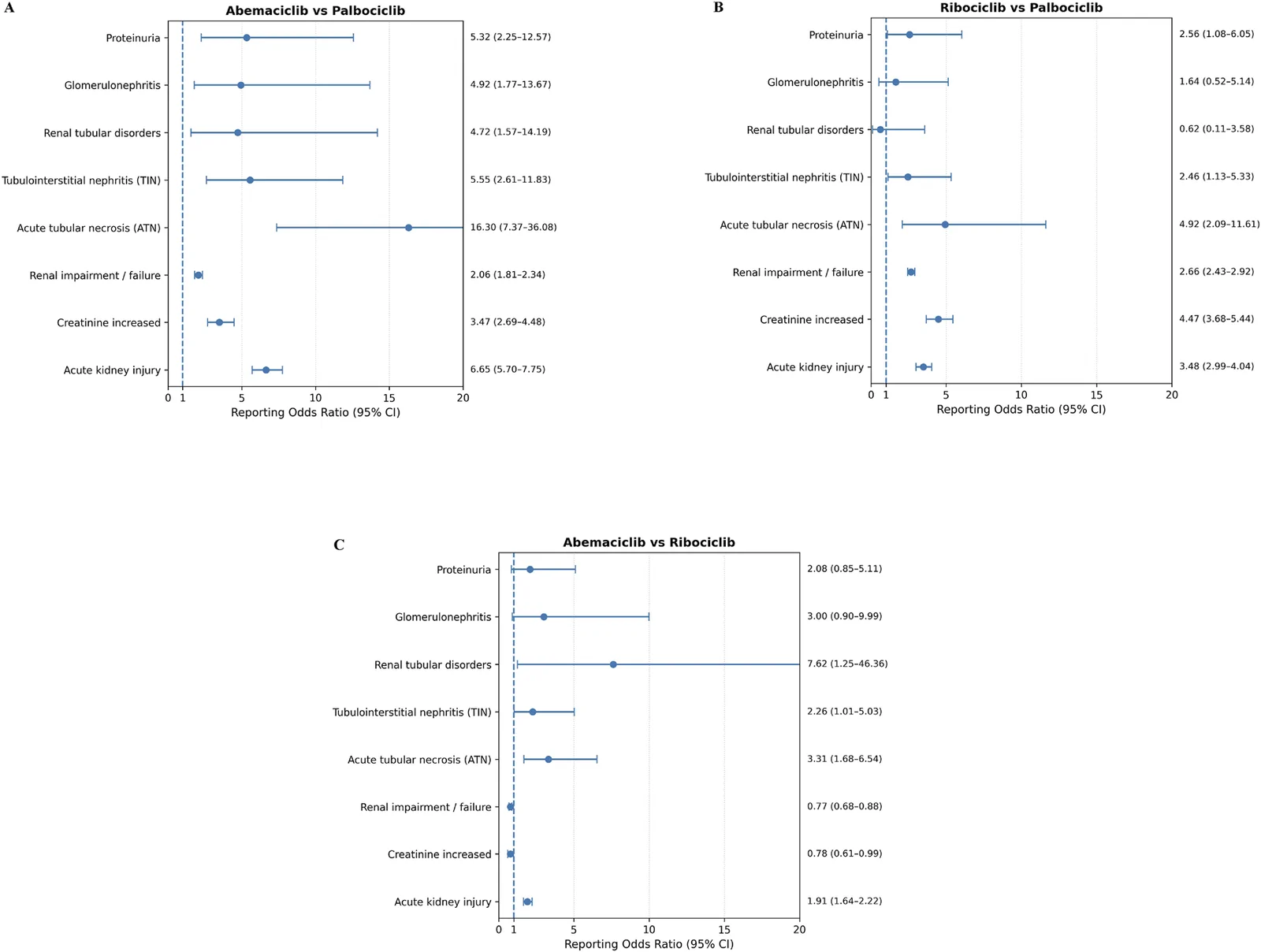
Pairwise disproportionality analyses of selected renal adverse events among CDK4/6 inhibitors. **(A)** Abemaciclib versus Palbociclib; **(B)** Ribociclib versus Palbociclib; and **(C)** Abemaciclib versus Ribociclib. Points represent reporting odds ratios (RORs), and error bars indicate 95% confidence intervals. The dashed vertical line indicates the null value (ROR = 1.0).

## 4 Discussion

In this pharmacovigilance study based on the FAERS database, we evaluated renal adverse events reported in association with the three currently available CDK4/6 inhibitors: abemaciclib, palbociclib, and ribociclib. By analyzing more than 135,000 deduplicated CDK4/6 inhibitor-related adverse event reports and identifying 5,360 renal adverse event reports, this study provides a real-world assessment of renal reporting patterns across individual CDK4/6 inhibitors. Rather than estimating incidence or absolute risk, our analysis was designed to characterize disproportionality signals and compare renal adverse event reporting profiles among these agents.

The main finding of this study is that renal adverse event reporting differed across individual CDK4/6 inhibitors. Abemaciclib demonstrated the highest reporting odds for acute kidney injury, followed by ribociclib, whereas palbociclib showed reduced reporting odds across all evaluated renal outcomes. In addition, ribociclib showed the strongest reporting associations with creatinine increased, decreased glomerular filtration rate, and electrolyte abnormalities, including hypokalemia, hyponatremia, and hypocalcemia. By contrast, abemaciclib was more prominently associated with reporting of acute kidney injury, acute tubular necrosis, tubulointerstitial nephritis, and renal tubular disorders. These findings suggest that renal adverse event profiles may not be uniform across CDK4/6 inhibitors and may reflect differences in pharmacologic properties, transporter interactions, off-target effects, and reporting patterns (Cui et al., 2024; Izzedine et al., 2025).

Serum creatinine elevations during CDK4/6 inhibitor therapy have been most extensively characterized with abemaciclib. Chappell et al. demonstrated that abemaciclib inhibits renal tubular transporters, including organic cation transporter 2 and multidrug and toxin extrusion transporters, leading to increased serum creatinine concentrations without changing measured glomerular filtration rate (Chappell et al., 2019). This mechanism supports the concept that some creatinine elevations observed during CDK4/6 inhibitor therapy may represent pseudo-nephrotoxicity rather than true kidney injury. However, our findings indicate that renal adverse event reporting extends beyond isolated creatinine elevation, particularly for abemaciclib and ribociclib, which showed disproportionate reporting for acute kidney injury.

The distinction between pseudo-acute kidney injury and true kidney injury is clinically important. While transporter-mediated creatinine elevations may not require treatment discontinuation, true renal injury can lead to hospitalization, treatment interruption, and potentially severe outcomes. Gupta et al. reported biopsy-confirmed acute kidney injury associated with CDK4/6 inhibitors and identified acute tubular injury, acute tubular necrosis, and interstitial nephritis as major histopathologic patterns (Gupta et al., 2022). These observations are consistent with our finding that acute tubular necrosis and tubulointerstitial nephritis were reported more frequently with abemaciclib than with palbociclib or ribociclib. Although FAERS does not provide biopsy confirmation or detailed clinical data, the consistency between pharmacovigilance signals and published clinicopathologic findings supports the clinical relevance of these events.

Ribociclib demonstrated particularly strong disproportionality signals for creatinine increased, decreased glomerular filtration rate, and electrolyte abnormalities. This pattern may reflect renal laboratory monitoring practices, drug-specific pharmacologic effects, or differences in the clinical contexts in which ribociclib is used. Electrolyte abnormalities are clinically relevant because they may coexist with renal dysfunction and may influence treatment safety, particularly in patients with advanced cancer, older age, comorbidities, or concomitant nephrotoxic medications (Murad et al., 2024). However, because FAERS lacks denominator data and detailed laboratory values, these findings should be interpreted as reporting signals rather than direct evidence of higher clinical risk.

The relatively lower reporting odds observed with palbociclib for several renal outcomes suggest fewer reported renal safety signals in the FAERS database. However, this interpretation requires caution. Differences in prescribing volume, time on the market, patient characteristics, reporting behavior, and comorbidity burden may influence disproportionality analyses. Palbociclib has been widely used for a longer period than other CDK4/6 inhibitors, and spontaneous reporting patterns may change over time as clinician awareness evolves (Köylü et al., 2025). Therefore, the lower reporting odds observed for palbociclib should not be interpreted as definitive evidence of lower nephrotoxicity risk.

The clinical outcomes observed in this study highlight the potential severity of renal adverse events associated with CDK4/6 inhibitors. Approximately two-thirds of renal adverse event reports were classified as serious, and hospitalization was reported in nearly one-third of cases. Death was reported in 12.0% of renal adverse event reports. These outcomes should not be interpreted as being directly caused by renal toxicity, because patients receiving CDK4/6 inhibitors often have advanced malignancy, comorbidities, and concomitant therapies (Köylü et al., 2025). Nevertheless, the high proportion of serious outcomes emphasizes the need for careful renal monitoring and early evaluation of kidney function abnormalities during CDK4/6 inhibitor therapy.

Our findings have several practical implications. First, clinicians should recognize that increases in serum creatinine during CDK4/6 inhibitor therapy may have different clinical meanings depending on the agent and clinical context. In patients receiving abemaciclib, isolated creatinine elevation may reflect transporter inhibition, but persistent or progressive renal dysfunction should prompt further evaluation. Second, renal adverse events should not be assumed to be benign laboratory abnormalities, particularly when accompanied by electrolyte disturbances, reduced urine output, proteinuria, systemic symptoms, or worsening estimated glomerular filtration rate. Third, alternative renal function markers such as cystatin C or measured glomerular filtration rate may help distinguish pseudo-nephrotoxicity from true kidney injury in selected patients (Vanhoutte and Sprangers, 2023; Errabelli et al., 2024).

This study has several limitations inherent to FAERS-based pharmacovigilance analyses. FAERS is a spontaneous reporting system and is subject to underreporting, overreporting, duplicate reporting, missing data, and reporting bias. The database does not provide reliable information on the total number of exposed patients, treatment duration, cumulative dose, baseline kidney function, comorbidities, concomitant nephrotoxic medications, or laboratory trajectories. Therefore, reporting odds ratios cannot be interpreted as incidence rates or relative risks. In addition, renal adverse events were identified using MedDRA Preferred Terms, and detailed clinical adjudication, biopsy confirmation, or causality assessment was not possible. Reported seriousness outcomes may reflect the overall clinical condition of the patient rather than renal toxicity itself. Finally, differences in drug approval dates, utilization patterns, and clinician awareness may influence reporting across individual CDK4/6 inhibitors. A further consideration relates to case ascertainment. Although the active ingredient search function of the FAERS Public Dashboard maps trade names and recognised synonyms to the corresponding generic name, drug entries submitted as misspelled, abbreviated or otherwise non-standardised free text may not be mapped successfully. A small number of eligible reports may therefore have been missed, and the counts presented here should be regarded as conservative estimates of the reports available in the database.

Despite these limitations, FAERS remains a valuable resource for detecting rare and clinically important safety signals that may not be fully captured in clinical trials. The present study provides one of the largest comparative pharmacovigilance assessments of renal adverse events associated with CDK4/6 inhibitors. Our findings suggest that renal adverse event reporting differs across abemaciclib, palbociclib, and ribociclib, with distinct patterns involving acute kidney injury, creatinine elevation, renal impairment, intrinsic renal injury, and electrolyte abnormalities. Further prospective studies with laboratory data, exposure denominators, and clinical adjudication are needed to validate these signals and clarify the mechanisms underlying CDK4/6 inhibitor-associated renal toxicity.

## 5 Conclusion

We present a large-scale pharmacovigilance analysis using FAERS data to characterize renal adverse event reporting associated with CDK4/6 inhibitors. Our findings demonstrate substantial differences in renal adverse event reporting patterns among abemaciclib, palbociclib, and ribociclib. Abemaciclib was more frequently associated with reporting of acute kidney injury, acute tubular necrosis and tubulointerstitial nephritis, whereas ribociclib demonstrated stronger reporting signals for creatinine elevation, decreased glomerular filtration rate, and electrolyte abnormalities. In contrast, palbociclib exhibited reduced disproportionality signals across all evaluated renal adverse events.

Although spontaneous reporting data cannot establish causality or estimate incidence, the observed reporting patterns suggest that renal toxicities associated with CDK4/6 inhibitors may be heterogeneous and potentially drug-specific. Given the substantial proportion of serious outcomes and hospitalizations observed in renal adverse event reports, clinicians should maintain awareness of both pseudo-nephrotoxicity and true kidney injury during CDK4/6 inhibitor therapy. Further prospective studies incorporating detailed clinical, laboratory, and histopathologic data are warranted to validate these findings, clarify underlying mechanisms, and optimize renal monitoring strategies for patients receiving CDK4/6 inhibitors.

## Data Availability

Publicly available datasets were analyzed in this study. This data can be found here: The data analyzed in this study were obtained from the U.S. Food and Drug Administration (FDA) Adverse Event Reporting System (FAERS), a publicly available pharmacovigilance database. Data can be accessed through the FDA FAERS Public Dashboard: https://www.fda.gov/drugs/drug-approvals-and-databases/fda-adverse-event-reporting-system-faers-database.

## Supplementary material

SUPPLEMENTARY TABLE S1 Renal adverse event preferred terms with ≥10 total reports across all CDK4/6 inhibitors (MedDRA Renal and Urinary Disorders System Organ Class).
