# Supplementary material for: Distinct renal adverse event profiles among CDK4/6 inhibitors: a comparative pharmacovigilance analysis
Source: Front Pharmacol. 2026 Sep 17;17:1917719. doi: 10.3389/fphar.2026.1917719 (PMC13627335; doi:10.3389/fphar.2026.1917719)
Supplement: Supplementary file 1 [file Table1.docx]

Supplementary Table S1. Renal adverse event preferred terms with ≥10 total reports across all CDK4/6 inhibitors (MedDRA Renal and Urinary Disorders System Organ Class)

| **Preferred Term (PT)** | **Abemaciclib n (%) N = 14,193** | **Palbociclib n (%) N = 91,359** | **Ribociclib n (%) N = 29,488** |
| --- | --- | --- | --- |
| Acute Kidney Injury | 293 (2.064) | 303 (0.332) | 321 (1.089) |
| Anuria | 11 (0.078) | 3 (0.003) | 8 (0.027) |
| Blood Creatinine Abnormal | 3 (0.021) | 20 (0.022) | 12 (0.041) |
| Blood Creatinine Decreased | 4 (0.028) | 10 (0.011) | 5 (0.017) |
| Blood Creatinine Increased | 82 (0.578) | 135 (0.148) | 217 (0.736) |
| Chronic Kidney Disease | 10 (0.070) | 110 (0.120) | 82 (0.278) |
| Creatinine Renal Clearance Decreased | 5 (0.035) | 11 (0.012) | 6 (0.020) |
| Creatinine Renal Clearance Increased | 1 (0.007) | 3 (0.003) | 8 (0.027) |
| End Stage Renal Disease | 1 (0.007) | 3 (0.003) | 9 (0.031) |
| Glomerular Filtration Rate Abnormal | 3 (0.021) | 3 (0.003) | 7 (0.024) |
| Glomerular Filtration Rate Decreased | 6 (0.042) | 50 (0.055) | 81 (0.275) |
| Hydronephrosis | 15 (0.106) | 58 (0.063) | 54 (0.183) |
| Kidney Infection | 2 (0.014) | 24 (0.026) | 18 (0.061) |
| Nephrolithiasis | 19 (0.134) | 162 (0.177) | 62 (0.210) |
| Nephropathy | 2 (0.014) | 21 (0.023) | 8 (0.027) |
| Nephropathy Toxic | 28 (0.197) | 16 (0.018) | 28 (0.095) |
| Oliguria | 1 (0.007) | 7 (0.008) | 9 (0.031) |
| Prerenal Failure | 1 (0.007) | 5 (0.005) | 8 (0.027) |
| Proteinuria | 9 (0.063) | 11 (0.012) | 9 (0.031) |
| Pyelonephritis | 2 (0.014) | 9 (0.010) | 7 (0.024) |
| Renal Colic | 3 (0.021) | 5 (0.005) | 3 (0.010) |
| Renal Cyst | 9 (0.063) | 38 (0.042) | 33 (0.112) |
| Renal Disorder | 88 (0.620) | 292 (0.320) | 127 (0.431) |
| Renal Failure | 127 (0.895) | 345 (0.378) | 362 (1.228) |
| Renal Function Test Abnormal | 3 (0.021) | 7 (0.008) | 4 (0.014) |
| Renal Impairment | 177 (1.247) | 540 (0.591) | 390 (1.323) |
| Renal Injury | 24 (0.169) | 8 (0.009) | 16 (0.054) |
| Renal Mass | 0 (0.000) | 7 (0.008) | 3 (0.010) |
| Renal Pain | 7 (0.049) | 35 (0.038) | 31 (0.105) |
| Renal Tubular Necrosis | 21 (0.148) | 8 (0.009) | 13 (0.044) |
| Renal Vein Thrombosis | 2 (0.014) | 0 (0.000) | 9 (0.031) |
| Tubulointerstitial Nephritis | 12 (0.085) | 14 (0.015) | 11 (0.037) |

*Values indicate the number of FAERS reports per preferred term. Multiple preferred terms may be reported per case. Percentages are calculated using the total number of deduplicated reports submitted for each drug as the denominator (abemaciclib, N = 14,193; palbociclib, N = 91,359; ribociclib, N = 29,488) and are shown to three decimal places because most preferred terms are rare. FAERS does not capture the number of patients actually exposed to each drug; these values therefore represent reporting proportions and must not be interpreted as incidence rates. Formal between-drug comparisons are provided as reporting odds ratios in Table 3.*
